# Kindergarten Affordances for Physical Activity and Preschoolers’ Motor and Social-Emotional Competence

**DOI:** 10.3390/children10020214

**Published:** 2023-01-25

**Authors:** Mariana Moreira, Guida Veiga, Frederico Lopes, Derek Hales, Carlos Luz, Rita Cordovil

**Affiliations:** 1 Interdisciplinary Center for the Study of Human Performance (CIPER), Faculdade de Motricidade Humana, University of Lisbon, 1499-002 Lisbon, Portugal; 2 Departamento de Desporto e Saúde, Escola de Saúde e Desenvolvimento Humano, Universidade de Évora, 7004-516 Évora, Portugal; 3 Comprehensive Health Research Centre (CHRC), University of Évora, 7004-516 Évora, Portugal; 4 Laboratory of Motor Behavior, Faculdade de Motricidade Humana, University of Lisbon, 1499-002 Lisbon, Portugal; 5 Center for Health Promotion and Disease Prevention, University of North Carolina at Chapel Hill, Chapel Hill, NC 27514, USA; 6 Research Center in Sports Performance, Recreation, Innovation and Technology (SPRINT), 4960-320 Melgaço, Portugal; 7 Escola Superior de Educação de Lisboa, Instituto Politécnico de Lisboa, 1549-003 Lisboa, Portugal; 8 Centro Interdisciplinar de Estudos Educacionais, 1549-003 Lisboa, Portugal

**Keywords:** kindergarten, physical activity, physical environment, social environment, quality, preschoolers, motor competence, social-emotional competence

## Abstract

This study examined the relationship between the quality of the kindergarten physical and social environment to promote physical activity (PA) and preschoolers’ motor and social-emotional competence. Two Portuguese kindergartens (Gondomar city) were selected from a pool of seventeen with an assessment of kindergarten PA best practices (one with high PA practices, the other with low). Thirty-six children (M = 4.42; SD = 1.00 years) without neuromotor disorders participated in this study. Motor and social-emotional competence were assessed with standardized motor skills tasks and parent report of child behaviors. Children from the kindergarten with higher compliance with PA best practices showed significantly better motor competence. No statistically significant differences were found for social-emotional competence scores. These findings emphasize the critical importance of kindergarten in promoting preschoolers’ motor competence by assuring a physical and social environment that enhances their PA practice. This is a particularly relevant concern for directors and teachers during the post-pandemic period, given the developmental delays and decreases in physical activity preschool children experienced across the pandemic period.

## 1. Introduction

Regular physical activity (PA) is vital to children’s health, wellbeing, and development [1,2,3], contributing significantly to motor and social-emotional competence during the preschool years (2–5 years). However, most preschoolers are not sufficiently active, with only 11% meeting or exceeding the more recent international guidelines for movement [4].

Providing children with PA opportunities when they are young optimizes the development of motor competence [5]. Kindergartens are an important environmental context for preschoolers’ development, where they should be given opportunities to practice a variety of movements and energetic play. That is, a PA-friendly kindergarten offers children conditions for engagement in physically active behaviors, which will have a positive influence on active and healthy lifestyle trajectories [5,6,7,8,9,10,11].

PA also plays a significant role in children’s social-emotional development [12]. When practicing PA, children activate physical sensations (e.g., racing heartbeat, rapid breathing, and high muscle tone) that are relevant to the emotional experience [13]. Exposure to, and familiarity with, these physical changes provide young children experience with feelings, which has been related to emotion-regulation skills [14]. For example, during rough and tumble play, which is often moderate or vigorous, children develop the skills needed to monitor their emotions and interactions with others, determining what “level” of play remains fun for everyone [13,15]. Scholars suggest that such emotional modulation during active play is necessary for children to develop the skills needed to maintain emotional control, rather than breaking down into aggression [13,15]. Moreover, physically active play provides many face-to-face opportunities and various types of interactions with others (e.g., sharing equipment, taking turns), which are essential elements in promoting the development of children’s social skills [16]. Finally, some studies also indicate that physical activity and motor and social-emotional competence contribute to preschoolers’ successful school readiness [17,18,19,20].

Gibson’s affordances theory [21] states that each environment has objects, places, surfaces, events, values, norms, rules, and other people that provide children with different action opportunities or affordances [21,22]. Current research supports the idea that these multidimensional characteristics, or affordances, of a kindergarten (i.e., physical attributes, written policies, and daily practices) account for a significant portion of the variability in preschoolers’ PA [2,8,23,24,25]. Actualizing the utility of these affordances occurs when children interact with the environment through a course of multidimensional invitations (physical, social-emotional, or symbolic) that co-emerge between the subject and the setting [22,26]. For this to happen, there has to be a mutual fit between the characteristics of children (e.g., motor skills, social needs, personal intentions) and the socio-physical attributes of the environment, which either promotes or restricts children’s fields of action through institutional norms, values, and rules [22,27,28]. While these affordances can promote activity [22], it is crucial to consider that other physical and social factors may interact to either facilitate (e.g., when written policies approve the children PA behaviors) or constrain (e.g., when the design of physical attributes do not meet the preschoolers’ PA development needs) children’s actions, therefore having the potential to positively or negatively impact children’s PA [22]. In this sense, kindergartens with supportive PA policies create social and cultural conditions nested within the physical environment, which foster children’s engagement with affordances that promote active play.

The influence of kindergarten PA affordances varies by setting (inside vs. outside), type (policy vs. environment), or behavioral target (reducing sedentary vs. increasing movement). In the outdoors, higher activity levels are often associated with time [2,29,30,31], the presence of open space, certain natural or artificial elements (e.g., grassy areas, trees, and shrubbery, cycling paths, markings, fixed equipment) [8,32,33], larger playgrounds [34], and the availability of sufficient portable equipment [31,34,35,36,37] while equipment and areas such as sandboxes, water toys, slides, and swings are associated with lower PA levels [31,32,33,38,39]. For indoor kindergarten spaces, children’s PA levels are positively associated with overall center/classroom size, having dedicated gyms or rooms with tumbling zones, open floor spaces, and some categories of portable equipment. These affordances seem to allow children to explore a wider range of movement patterns (i.e., kneel, crawl, walk, hop, run), promote energetic play, and have been shown to reduce the physical contacts between children that often slow or prevent running or chasing games [32,39,40,41,42,43,44]. Lastly, studies show that factors such as formalized PA written policies (i.e., daily teacher-facilitated PA [37], limited screen-viewing [45], teachers’ PA knowledge [31], staff education and training [46], and staff behavior and encouragement [24]) have a positive impact on children’s PA [2,29,47,48]. Thus, a kindergarten can promote children’s movement and physical activity play through PA affordances, that is, through the physical environment properties and the social environment (i.e., written policies and daily practices).

Many of these PA affordances have also been related to children’s motor and social-emotional competence. For example, children who spend more time outdoors tend to have better overall motor competence. If the outdoor area includes a diversity of natural elements, specific motor skill scores such as balance and coordination are higher [49]. In addition, time spent in green spaces outdoors positively impacts social-emotional competencies [50], especially self-esteem, confidence, emotional and behavioral regulatory skills [51], problem-solving [49,52,53], children’s empathy [49], and pro-social behavior [38,54]. Furthermore, the implementation of teacher-led group physical education activities can be a way to improve preschoolers’ social skills [16]. For inside spaces, density (children per m^2^) has been found to influence both motor- and social-competence. In kindergartens/classrooms with a higher density (i.e., more crowded), children are less likely to use energetic play and more likely to exhibit aggressiveness, withdrawal, and hyperactivity [46,55].

To date, only one study has assessed the relationship between preschoolers’ PA and social-emotional functioning, showing that being physically active had a positive relationship with self and social awareness, relationship skills, and optimistic thinking [12]. However, to the best of our knowledge, no study has yet examined the specific relationship between the PA affordances in kindergarten and preschoolers’ motor and social-emotional competence. For this reason, the purpose of this research is to examine the difference in children’s motor-competence and social-emotional competence between two kindergartens with known differences in PA affordances. We hypothesized that preschooler’s motor- and social competences will be significantly different between a kindergarten with higher and lower PA affordances. Specifically, in a setting with better PA best-practice compliance, children will have higher scores on the motor competence assessment and the strengths and difficulties questionnaire [12,16,49,50].

## 2. Materials and Methods

### 2.1. Procedures

The study was conducting in two stages. In stage 1, two kindergartens with divergent PA affordances were identified (KG-high and KG-low). Following this (stage 2), the motor skills and social-emotional characteristics of children from two classrooms (one from KG-high, one from KG-low) were measured and compared. All procedures were carried out following the 1964 Helsinki declaration and its later amendments. The Ethics Committee of the Faculty of Human Kinetics at The University of Lisbon approved all study procedures (CEIF Approval Number: 26/2019).

During stage 1, directors from half of the kindergartens in Gondomar city, Portugal, were invited by telephone and email to a face-to-face meeting, where the researcher explained the purpose of the study and its procedures. Directors who agreed to participate in this study, filled out a demographics questionnaire and were asked to have 1 or 2 teachers complete a self-report assessment of the programs PA affordances. From this information the five kindergartens with the highest and lowest scores were identified and allocated to the KG-high and KG-low quality group. During stage 2, only one kindergarten from each group (KG-high, KG-low) was recruited to participate. During recruitment, kindergartens with the highest and lowest scores were approached in order until one from each group agreed to take part. The use of just two kindergartens was mainly due to the restrictions required in the kindergartens due to COVID-19. Parents were provided information about the project and asked to participate. Parents who agreed completed surveys with information about demographic variables (e.g., age, sex, parents’ job, and education level), children’s routines regarding the time spent in kindergarten and on directed and non-directed PA outside the kindergarten, and children social-emotional functioning.

After obtaining consent from these two kindergartens’ directors and the parents of children, and before starting the data collection, the first author spent one week in each kindergarten to get familiar with their routines and to create a relationship with the teachers and children. The idea was to guarantee that everyone acted naturally during data collection, not being influenced by the novelty of the researcher’s presence. Following this period, the motor skills assessments were conducted with small groups of children. The data collection took place between December 2020 and May 2021, with an interruption between January 2021 and February 2021 due to the COVID-19 lockdown in Portugal.

### 2.2. Sample and Participants

During stage 1, about half of the kindergartens in Gondomar city (Porto area in Portugal) were approached (n = 36) to participate. Of these, 19 directors consented and 17 completed all study measurement. The primary reason directors refused were COVID-19 restrictions. During stage 2, one class from KG-high and one from KG-low were chosen based on their teachers’ availability to participate in the study. Thirty-six parents/guardians and their children (seventeen boys and nineteen girls) were recruited from these two classrooms. Children had no known motor or cognitive disorders.

### 2.3. Instruments

#### 2.3.1. Kindergarten Affordances for Physical Activity

The Environment and Policy Assessment and Observation-Self-Report (EPAO-sr) [11] was used to assess and quantify the environmental and social setting and policies in the kindergarten that promote PA (see Moreira et al. [56] for the Portuguese version). Following the instrument’s procedures, three surveys were administered: the director general survey (to assess written policies regarding time and space for PA, training for staff, and parental education), teacher general survey (to assess the features of the physical environment that promote PA and active play) and teacher daily survey (to assess daily practices and time to promote PA). Items from the three surveys were combined and used to score 13 sub-components (see Table 1). Each subcomponent is scored on a 0 to 3 scale, with a score of 3 indicating PA best practice in this area was met or exceeded. The EPAO-sr PA total score was computed as a simple sum of the 13 sub-component scores and could range from 0 (lower compliance) to 39 (higher compliance).

#### 2.3.2. Motor Competence

Children’s motor competence was measured with the motor competence assessment (MCA) [57,58]. The MCA includes three subscales: stability skills (lateral jumps, shifting platforms), locomotor skills (standing long jump, 4 × 10 m shuttle run), and manipulative skills (ball kicking velocity, ball throwing velocity). All motor tests are quantitative and do not have a marked developmental (age) ceiling effect. Children performed all MCA tests in small groups (about three children for each task). Group sessions lasted for approximately 15 min [57]. In total, children from each kindergarten spent 90 min completing MCA tests over a one-week period. The data collection followed standard procedures: (a) a proficient demonstration of each test technique was provided, along with a verbal explanation; (b) every child tried each task once before the assessment; (c) the instructions emphasized that children should try to perform the task at their maximum potential (e.g., “as fast as possible” for the stability tests and 4 × 10 shuttle run; “as far as possible” for the standing long jump; and “as hard as possible” for the manipulative tests); (d) motivational feedback was given, but no verbal feedback on skill performance was provided. The MCA testing took place at each kindergarten in a designated area with enough space for a given MCA assessment. To compute each child’s motor competence scores, results in each test of the MCA were transformed into a percentile value relative to age and sex, according to the MCA norms. Total MCA was calculated by the average of the three subscale percentiles [58]. In addition, children were subdivided into tertile groups of motor competence (high, average and low), according to their percentile score on the total MCA and each subscale (stability, locomotor, manipulative) [59].

#### 2.3.3. Social-Emotional Competence

Social-emotional competence was obtained through pro-social behavior, peer problems, and externalizing behaviors, measured with the Portuguese version for parents of the strengths and difficulties questionnaire [60]. This questionnaire has 25 items grouped in five subscales of five items each (“hyperactivity”, “emotional symptoms”, “conduct problems”, “peer problems”, and “pro-social behavior”). Parents rated each item about their child’s behavior (e.g., “is sensitive to the feelings of others”) on a 3-point scale with 0 (not true) and 2 (certainly true). In this study, the Cronbach’s alpha coefficient was acceptable for the sub-scale “peer problems” (α = 0.60) and good for the “pro-social behavior scale” (α = 0.75). Following Wiefferink et al. [61], the “hyperactivity” and “conduct problems” sub scales were scored as one composite scale: externalizing behaviors. The internal consistency of this composite scale was good (Cronbach’s alpha coefficient = 0.68). Scale scores were computed by averaging items scores.

### 2.4. Data Analysis

Means, standard deviations, frequencies, and all analysis were conducted using SPSS (v25). To find centers with high and low affordances for PA, EPAO-sr total scores from the 17 kindergartens were used to identify the 5 lowest and 5 highest scoring centers. Due to the normality of the motor competence data, independent samples T-tests were used to compare mean motor competence scores between children in KG-high and KG-low, while due to the non-normality of the social-emotional data, Mann–Whitney tests were used to test differences in social-emotional competence scores.

Distributions and frequencies of children in the high, average, and low MCA groups were compared across the two kindergartens. Finally, social-emotional competence scores, exposure to directed PA, and time in kindergarten each day were compared across the MCA total score tertiles.

## 3. Results

### 3.1. Stage 1. Select and Identify Centers with High and Low Affordances for PA

Of the 19 kindergarten directors who consented, 17 provided completed EPAO-sr and demographics information. For these centers (n = 17), average EPAO-sr total score was 19.77 + 4.61, with centers scoring 16 or less in the lower third and centers scoring 23 or above in the highest third. The two kindergartens who volunteered to participate had differences in 62% of the EPAO-sr sub-component scores (8 of 13), with total scores approximately 11 points higher (69%) in KG-high compared to KG-low. The EPAO-sr total PA score for the KG-high program was highest out of all 17 programs, with the score for KG-low being in the bottom third of all scores. Total and sub-components scores for the EPAO-sr for each kindergarten are shown in Table 1.

KG-high was from the public sector. It hosted just one preschool class with nineteen preschoolers, one teacher, and three teaching assistants. This kindergarten was in a poorer neighborhood and its building was on the ground floor of a block of apartments. It offered one classroom for indoor pedagogical activities and one multipurpose room, where motor skills classes occurred. The floor of the outdoor usable area was all in cement, with one floor marking for hopscotch. There was a roof to protect part of the playground from the rain and intense sun. No fixed playground equipment was offered. Small, loose materials and toys were available for children to use, most were for low-activity dramatic and symbolic play (e.g., baby dolls, stuffed animals, cars, dolls, musical instruments). On some occasions, hula-hoop and small balls were also provided for children’s play in the multipurpose room and in the playground.

KG-low was from the private sector. It had sixty-six infants, toddlers, preschoolers, and first-grade children, enrolled with seven teachers and four teaching assistants. It was in an upper-class neighborhood in a two-story independent building. The preschoolers were subdivided in two different classrooms, with 19 children per class, and each had motor skills classes on a multipurpose room. In the outdoor space there were large grass areas and small cement areas, with various play areas, such as a tank for playing with dirt and mud (when it rains), a big sand box, a grassy area, and fixed swings. Some loose parts (e.g., shovels, buckets, cups, balls), and other portable equipment, such as a bike and a slide, were accessible to the children. There was no roof protection in this outdoor space.

In Table 2 it is possible to see the dimensions of the kindergarten physical environment indoor and outdoor areas.

KG-high met best practice in 23% of the affordances measured with the EPAO-sr, daily PA practices, screen time policy, and the outdoor play and learning written policies. Both kindergartens showed similar quality indicators in the PA written policies, PA time, and outdoor playtime provided. KG-low met 0% of best practice indicators, with lower quality indicators concerning promoting teachers’ professional development in PA, screen time, outdoor play and learning, and play equipment offered indoors.

A detailed analysis of the EPAO-sr items indicated that children in both kindergartens spent about 30 min/day in active play indoors. However, children in KG-high had about 72 min per day of active play outdoors, compared to only 55 min/day in KG-low. According to teacher reports, children did not engage in vigorous activities indoors or outdoors in either kindergarten, but higher general activity levels were reported in KG-high compared to KG-low (i.e., mostly moderate walking fast and skipping vs walking slowly and marching). A structured PA lesson was offered weekly in both kindergartens by a PA specialized teacher, so most days children did not have 60 min of adult-led PA. Both kindergartens have an indoor multipurpose room available for PA, although this space was smaller in KG-high, limiting the possibilities for vigorous PA. Overall, there was a greater variety of indoor equipment in KG-high than in KG-low (e.g., portable tunnels, balance toys, mini tramps). In the outdoor environment, KG-high had few natural elements and fixed equipment, but did have a roof over the outside space, providing the necessary conditions to go outside on rainy days. Both kindergartens offer training sessions for parents in PA and in outdoor play and learning twice a year. KG-high also provides teacher training in PA, outdoor play and learning, and screen time.

### 3.2. Stage 2. Comparison of Motor Skills and Social-Emotional Competence

A total of 17 children (10 boys and 7 girls) from KG-high (M = 4.41 ± 0.71 years) and 19 children (7 boys and 12 girls) from KG-low (M = 4.44 ± 1.26 years) were recruited and measured for stage 2 of this study. Families in KG-low were mainly middle class (64.7%), and upper class in KG-high (73.3%). Children’s time spent in kindergarten and in PA out of kindergarten are presented in Table 3. Parents reported that children from KG-low tended to spend more time in kindergarten each and in directed PA out of kindergarten, but less time in non-directed PA.

MCA scores were 30%–70% higher for children attending KG-high compared to KG-low. Results indicated these differences were statistically significant and had a large effect size for total motor competence (t (34) = −3.63, *p* = 0.004, d = 1.21) and manipulative skills (t (34) = −3.65, *p* = 0.001, d = 1.22). A summary of MCA scores and comparisons between kindergartens is shown in Table 3.

**Figure 1:**
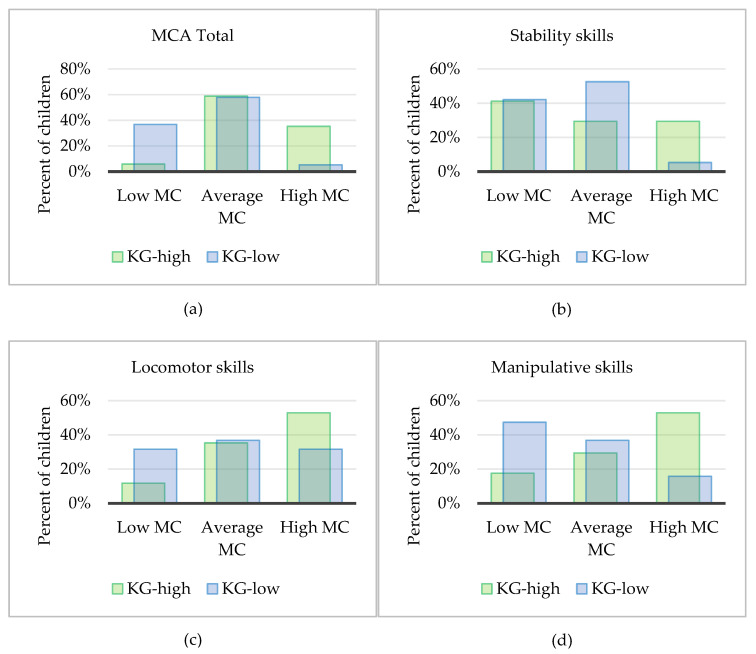
Percent of children from KG-high and KG-low by (**a**) Motor Competence Group for total, (**b**) stability, (**c**) locomotion, and (**d**) manipulative MCA.

Figure 1 shows the distribution of children from both kindergartens across the low, average, and high MC proficiency groups, for each MCA domain. The percentage of children in the high MC group was greater in KG-high than in KG-low for MCA total (35% vs. 5%), stability skills (29% vs. 5%), locomotion (53% vs. 33%), and manipulative skills (53 % vs. 16 %).

Results of the social-emotional competence assessments are presented in Table 3. Mann–Whitney tests revealed no significant differences in social-emotional competence between children from the two kindergartens.

## 4. Discussion

Kindergarten is a critical context for children’s daily routines and behaviors [6]. The findings of this study suggest that children in a PA-friendly kindergarten (meeting more best practices) had better motor competence. These findings are in line with previous research [9,29,31,63] and support the idea that both the characteristics of the kindergarten’s physical space, policies, and daily practices can facilitate, or inhibit, factors that influence children’s motor competence [22]. These aspects are interrelated and dependent on each other, since children’s increase and diversity of fields of action for active play and physical activity, both with and without adult prompting, depend on the material and social features and layers of the environment [21]. A play setting conducive to PA that is accompanied by a flexible and permissive approach by adult providers is fundamental for children to engage in enriched active play opportunities, which is one of the key aspects for improving both PA [64] and motor competence. Therefore, we believe that differences between the two centers studied influence the types and amounts of PA children chose from day to day [28,41].

Small studies and pilot work, while limited in generalizability, are necessary stepping stones for making progress in any area of research. These efforts often draw attention to results that need further investigation, help identify new or overlooked practical and theoretical questions, and aid in refining methodologies and procedures that can be pursued in larger trials and more costly interventions. Our study provides a unique model for examining the influence of PA affordance on preschoolers’ motor and social-emotional competence. It is a common approach to focus on groups with known differences, however it is rarely used to examine the impact of kindergarten environments on behavior. Although our work is limited by sample size, known and unknown group differences, and the subjectivity of some measures, the significant differences in MCA found between children from the two kindergartens (one with high and the other with low PA affordances), should not be dismissed. Such results reinforce the theoretical approach that a child-friendly play and learning environment in early childhood educational settings promotes the opportunity for children to engage in diversified PA actions [64]. Nevertheless, the influence of other characteristics should be acknowledged in future studies, such as the type of program, gender distribution, family PA, among others.

This work highlights the need to design future studies in this area to explore a number of important questions, for example (1) which PA affordances influence each area of motor competence, and (2) is the relationship between meeting best practice scores and motor competence linear? In this study, we compared a center meeting 0% of best practices to one meeting 23%. While differences in all areas of motor competence were clinically meaningful, we cannot determine if effects would be smaller if only 10% of best practice were met, or larger if 50% of the PA affordances were provided. As the field moves forward, distinguishing between a linear and threshold relationship between affordances and motor skills, or physical activity, will be beneficial.

In addition, the present study showed that children who attended the kindergarten with the smaller playground had significantly better manipulative skills (the only statistically significant MC component). This finding is in line with a previous study [65] which explored the influence of different sizes of free play areas in kindergarten on preschoolers’ motor competence, showing that children with a smaller kindergarten play area had better object control skills. In our study, children with better object control (and the small play area) also had a greater availability of balls and other materials that encourage children to practice manipulative skills (e.g., hoops, scarves, buckets, dolls, mini-cars). We also hypothesized that the smaller area and surface material (cement) prompted teachers to promote small-scale object manipulation over expansive and energetic movements (e.g., running, jumping, sports) [22] in order to prevent injury and limit agitation, or negative encounters, among the children [55]. Furthermore, a cement surface can invite additional practice “tracking and catching” through bounce/dribble ball manipulation. In our future work we plan to examine this by including direct observation of teacher and child behaviors in spaces of various size and through measurement of staff perceptions of appropriate activities within the kindergarten space.

Although previous studies [38,49,50,51,52,53] have found that certain kindergarten physical elements and pedagogical practices (PA enhancers or inhibitors) are related to the child’s social-emotional competence, the results of this study highlight the importance of examining this relationship more holistically. That is, future studies should consider the influence of other variables present in kindergartens and the home which are not directly linked to PA promotion (e.g., how the teacher manages children’s interactions and emotions, parenting style). Although engaging in PA is a significant opportunity to gain awareness of bodily sensations and develop social-emotional competence [13], in our sample, children attending the kindergarten that favored PA did not have better social emotional competence scores. Children’s emotion socialization is complex and includes significant incidental learning (i.e., observing or overhearing others, reading, or watching cartoons) [66]. Therefore, these findings suggest that social-emotional development depends as much on the social dimension of the children’s environments as the physical dimension. Future studies will need to directly and systematically access children’s play behavior both indoors and outdoors, with a focus on associations between different types of play, child–child, and child–adult interactions across the day.

In this work, the researcher’s presence in the kindergarten, five days before starting data collection, was fundamental to confirm in loco the information collected by the EPAO surveys. Unfortunately, we were unable to utilize this time to observe other child interactions and had to rely on a subjective parent report of social emotional competence and a teacher report of average PA intensity during inside and outside time. These methods were less than ideal, and future studies should strive to complement self-reported data with more objective measures of children’s PA (e.g., assessment tasks, accelerometers, pedometers) and observations of staff behaviors. Furthermore, we suggest that future studies analyze whether the application of specific PA-promoting practices conducted by specialists (i.e., physical education teachers; psychomotor therapists) impact preschoolers’ motor and social-emotional competence.

## 5. Conclusions

The outcomes of this study show that higher compliance with best practice indicators to promote PA is related to preschoolers’ motor competence. The absence of a relationship between the kindergarten PA affordances and social-emotional competence points to the need to analyze this relationship more holistically. Our exploratory findings reinforce the need for preschool directors and teachers to collaborate and work on measuring, reviewing, and monitoring policies and practices that foster PA in physical and social environments [2,11]. Such endeavors are crucial to help and support children achieve adequate PA levels, thus contributing to positive motor and social-emotional development [2,9,50] and to improved school readiness [19].

In the aftermath of the COVID-19 pandemic, which negatively impacted preschoolers’ PA behavior, development, and well-being [67,68], the present research brings to light some relevant reflections on the role of kindergarten policy leaders and decision-makers, namely, the roles of directors’ and teachers’ as active promoters or inhibitors of PA healthy routines and environments for children in their care.

## Figures and Tables

**Table 1:** Compliance with EPAO-sr best practices to promote PA in each kindergarten.

	Kindergarten ^a^	
Sub-Components	KG-High	KG-Low
1. PA time provided	2	2
2. Indoor play equipment	2	1
3. Daily PA practices	3	2
4. PA teacher professional development	2	1
5. PA written policy	2	2
6. Screen time	1	1
7. Daily screen time practices	2	2
8. Screen Time teacher professional development	2	0
9. Screen Time policy	3	1
10. Outdoor playtime	2	2
11. Outdoor play environment	1	2
12. Outdoor play and learning teacher professional development	2	0
13. Outdoor play and learning written policies	3	2
PA final score	27.37	15.73
Percent of components meeting “best practice” criteria	23.1%	0.0%

^a^ Higher scores indicate better compliance with best practice standard. Score of 3 = meeting best practice.

**Table 2:** Characterization of the kindergarten physical environment indoor and outdoor spaces.

Physical Environment Dimensions ^a^	KG-High	KG-Low
Total gross area of the building	78.0	315.7
Classroom area	44.0	48.5
Multipurpose room (for motor skills classes)	36.0	56.7
Outdoor play area	25.0	1697.0

^a^ All the presented dimensions are in m^2^.

**Table 3:** Differences between KG-high (n = 17) and KG-low (n = 19) for children age, hours spent in kindergarten, hours in non-adult and adult led PA out of the kindergarten, and motor- and social-emotional competence outcomes.

Outcome	KG-High		KG-Low			
	Mean	SD	Mean	SD	Test, *p*	Cohen’s d ^c^
Children Age	4.41	0.71	4.44	1.26	-	-
Kindergarten time ^ab^	5.29	2.57	7.90	0.16	U = 11.50, *p* < 0.001	-
Non-led PA out of kindergarten ^ab^	2.86	0.77	2.26	0.96	U = 92.50, *p* = 0.052	-
Led PA out of kindergarten ^ab^	1.14	0.77	1.60	0.63	U = 92.5, *p* = 0.189	-
**Motor Competence**						
MCA total score (percentile)	60.53	16.84	40.05	16.94	t (0.275) = −3.63, *p*= 0.001	1.21
Stability (percentile)	45.02	21.07	34.20	21.71	t (1.53) = −1.33, *p* = 0.192	0.44
Locomotor (percentile)	65.34	23.41	49.90	27.58	t (0.303) = −1.80, *p* = 0.081	0.60
Manipulative (percentile)	65.87	24.43	38.05	21.35	t (0.371) = −3.65, *p* = 0.001	1.22
**Social-Emotional Competence**						
Peer Problems (0–10) ^b^	0.25	0.24	0.17	0.34	U = 93.0, *p* = 0.161	0.27
Pro-social behavior (0–10) ^b^	1.63	0.37	1.81	0.19	U = 95.5, *p* = 0.211	0.25
Externalizing Behavior (0–10) ^b^	0.74	0.28	0.65	0.27	U = 94.5, *p* = 0.207	0.26

^a^ hours/per day; ^b^ Mann–Whitney test, PA = Physical Activity, ^c^ Effect size interpretation was based on benchmarks suggested by Cohen [62] as small (d ≤ 0.20), medium (0.20 < d < 0.80) and large (d ≥ 0.80).

## Data Availability

The data presented in this study are available on request from the corresponding author. The data are not publicly available due to privacy or ethical restrictions.
